# Constraint aware and uncertainty informed QSAR modeling of Tetrahymena pyriformis toxicity using physicochemical descriptors

**DOI:** 10.1038/s41598-026-50786-7

**Published:** 2026-04-29

**Authors:** El-Sayed Khafagy, Amr Selim Abu Lila, Ahmed Al Saqr, Mahboubeh Pishnamazi

**Affiliations:** 1 Department of Pharmaceutics, College of Pharmacy, Prince Sattam bin Abdulaziz University, Al-kharj, 11942 Saudi Arabia; 2 Department of Pharmaceutics, College of Pharmacy, University of Ha’il, Ha’il, 81442 Saudi Arabia; 3 Institute of Research and Development, Duy Tan University, Da Nang, Vietnam; 4 School of Engineering & Technology, Duy Tan University, Da Nang, Vietnam

**Keywords:** Aquatic toxicity prediction, Tetrahymena pyriformis, Physicochemical descriptors, Predictive uncertainty quantification, Monotonicity-constrained learning, Chemistry, Computational biology and bioinformatics, Mathematics and computing

## Abstract

Conventional Quantitative Structure Activity Relationship (QSAR) models for predicting aquatic toxicity typically focus on accuracy at the expense of uncertainty awareness, physicochemical consistency, and chemical similarity leakage risks. Hence, the predictions are unstable when tested over different chemical spaces. This paper presents a machine-learning framework to predict the toxicity of *Tetrahymena pyriformis* based on a dataset of 1792 organic compounds whose 40-h growth inhibition (IGC50) values were experimentally determined and converted to pIGC50 for the models. The goal is to generate trustworthy and explainable results by using only eight molecular descriptors that are physicochemically relevant as model inputs. The proposed framework combines monotonicity-constrained learning, predictive uncertainty estimation, and intervention-inspired sensitivity analysis in one pipeline. The model’s reliability has been tested by cluster-aware data splitting, cross-validation, external testing, Y-scrambling, and applicability-domain assessment. The final method is capable of producing consistent, explainable, and risk-aware predictions and can be used as a reliability-centered basis for QSAR-initiated aquatic toxicity screening of chemically diverse compounds.

**Supplementary Information:**

The online version contains supplementary material available at 10.1038/s41598-026-50786-7.

## Introduction

The industrial and consumer product landscape is driven by the rise in sustainable and innovation-led chemistry, which has promising applications in many fields (e.g., health, technology, energy, etc.). The quantity of commercial chemicals and the pace of worldwide chemistry research are clearly correlated^1,2^, with a notable rise in the production, marketing, and eventual environmental identification of synthetic compounds. The dearth of information regarding the possible health and environmental hazards of most of these chemicals is a major cause for concern; only about 11% of chemical substances marketed in the US and the EU have experimental aquatic toxicity data available, 1% have bioconcentration data, and 0.2% have persistence data^3^. This emphasizes the necessity of thorough and efficient risk evaluations in addition to the appropriate handling of all novel chemical compounds in order to avoid or reduce any possible negative impacts on the environment and human health^4,5^. In order to close data gaps, computer-aided techniques, specifically, read-across and quantitative structure-activity relationships, or QSARs, are valuable and crucial tools to supplement conventional acute toxicity assessment^6–8^. As more organic compounds are produced and released into the environment, organic products in aquatic environments pose a threat to aquatic life and ecological systems, particularly to humans, by causing illnesses, including cancer and gene damage^8^. Before putting chemicals onto the market, toxicity evaluation is crucial for the chemical industry. Chemical toxicity can be predicted using QSTR/QSAR models. With regard to Tetrahymena pyriformis, a number of QSAR and machine learning models have been created to forecast the 50% growth inhibition concentration of compounds, or pIGC50. These include neural network models with minimum descriptors, support vector regression techniques for aromatic chemicals, and random forest models for big datasets of pIGC50 values that demonstrated great predictive ability. Despite these advancements, the majority of current models do not quantify predictive uncertainty, enforce physicochemical restrictions, or discover causal descriptor links; instead, they concentrate on prediction accuracy^9^. QSTRs have grown in importance in forecasting the toxicity of compounds, particularly those that have not been produced, because of their cheaper cost and faster speed. Tetrahymena pyriformis has been the subject of several toxicity studies over the years because of its many beneficial characteristics, including the ability to grow in a lab, a short life cycle, high sensitivity to toxins, and sensitivity comparable to that of human cell culture^10^. Establishing connections between chemical structure descriptors and biological activities (such toxicity) or qualities is the goal of QSARs, an in silico toxicology technique. It is implicitly assumed that compounds with similar structures should exhibit similar behaviors and characteristics, and that patterns within molecular groupings may be recognized and predicted^11^.

Recent research highlights that QSAR and machine-learning models, which use molecular descriptor-based representations, show strong predictive performance for aquatic toxicity endpoints. Descriptor-driven QSAR approaches have demonstrated the ability to predict concentration-based toxicity measures such as EC50 and LC50 across various chemical classes, indicating that low-dimensional physicochemical descriptor still provide a wealth of information for toxicity evaluation. An example includes the identification of a small set of interpretable descriptors that could consistently predict agrochemical toxicity^12^, while ensemble ML models were able to classify high-accuracy acute toxicity to aquatic organisms^13^. In the same way, descriptor-based QSAR models have also been effectively utilized for toxicity prediction of *Tetrahymena pyriformis*^14^. Recent publications show that targeting maximum precision with QSAR modeling can be considered feasible and profitable for applications; however, those models generally lack consideration of predictive uncertainty and physicochemical consistency. In order to improve regulatory applicability and mechanistic relevance, research combining QSAR modeling with read-across and similar methods can be seen. Hybrid QSAR, q-RASAR models hybridize statistical and mechanistic aspects to achieve better prioritization of developmental toxicity^15^, while QSAR and read-across workflows in line with OECD standards have shown to be dependable for chronic aquatic toxicity prediction^16^.

Additionally, best-practice guidelines highlight the significance of the model’s transparency, the evaluation of the applicability domain, and its reliability in a regulatory setting^17^. Nevertheless, these methods are still largely based on correlation and rarely integrate reasoning in the form of interventions about the effect of changes in descriptors on toxicity outcomes. Recently, there has been a noticeable change in emphasis toward model interpretability in toxicity modeling efforts. Descriptor-based ML models have been used together with post-hoc interpretation methods, such as SHAP, to unveil chemical features associated with toxicity^18,19^. Furthermore, interpretable structural parameter models have demonstrated a relatively good fish LC50 prediction performance^20^. These methods not only make the modeling processes more transparent but also, they mainly focus on analyzing the correlations that have been learned without requiring that the model’s behavior be physicochemically consistent. In parallel, advanced ML techniques have recently taken multimodal learning approaches to increase the predictive power of toxicity models^21^.

While the principles of model validation, applicability domain definition, and mechanistic interpretation have been well established within the QSAR community, particularly following the OECD guidelines, their integrated implementation within low-dimensional physicochemical descriptor-based machine learning frameworks remains an area of ongoing development. In this context, the present study aims to combine physicochemical consistency, uncertainty awareness, and controlled interpretability within a unified modeling pipeline, rather than introducing entirely new QSAR principles.

## Dataset

### Data curation and integrity control

Toxicity data on *Tetrahymena pyriformis* were taken from the curated dataset of Fang et al.^22^ that contained 1792 organic compounds with IGC₅₀ values determined experimentally after 40 h exposure. The endpoint was transformed into pIGC₅₀ (log IGC₅₀) to increase modeling stability. Only organic compounds that could be described with descriptors were kept, whereas mixtures, inorganic species, and entries that had inconsistent structural information were removed so that the descriptors would be reliable. SMILES strings were normalized and checked, and duplicates were removed before the descriptors were calculated through SMILES-based deduplication to prevent an overly optimistic bias induced by chemically redundant compounds. Following preprocessing, the whole dataset of 1792 compounds was available for modeling. The data were next split into a training set (80%, 1434 compounds) and an external test set (20%, 358 compounds) such that the test samples were not part of model development^5,23^.

The external test set was constructed using a cluster-aware partitioning strategy designed to reduce similarity-driven information leakage. Clustering was performed in the initial filtered descriptor space, after removing low-variance and highly correlated descriptors but prior to the final selection of eight descriptors. Principal component analysis (PCA) was first applied to this descriptor space, followed by k-means clustering to group chemically similar compounds. Entire clusters were then assigned to the test set to ensure structural dissimilarity between training and testing samples. While this approach shares conceptual similarities with leave-class-out validation, it does not rely on predefined chemical classes but instead derives data-driven groupings based on descriptor similarity. This enables a more flexible and continuous representation of chemical space.

### Descriptor calculation and physicochemical screening

The study calculated molecular descriptors using well-known cheminformatics tools that covered a wide range of molecular features, such as constitutional, topological, functional-group, and physicochemical properties. To avoid unstable model behavior, the descriptors that had missing values, near-constant behavior, or were independent or redundant descriptors. The initial descriptor pool consisted of approximately 1400 two-dimensional molecular descriptors computed using PaDEL-Descriptor. Following preprocessing steps, including removal of descriptors with missing values, low variance, and high inter-correlation (|r| > 0.9), the descriptor space was reduced to approximately 200 stable and informative features. The final selection of eight descriptors was performed using supervised ranking within the training data. Among these, descriptors labeled A and B do not correspond to single predefined PaDEL descriptors but represent normalized composite indices derived from combinations of atom-level connectivity and electronic distribution features. These composite descriptors were constructed to capture aggregated physicochemical effects not represented by individual descriptors alone. Their inclusion enhances the representation of structural and electronic characteristics within the reduced descriptor space.

All descriptor calculations and model implementations were performed in Python (version 3.10). Key libraries included Scikit-learn (v1.3), XGBoost-compatible gradient boosting frameworks (v1.7), and PyTorch (v2.0) for Bayesian neural network implementation, with probabilistic layers constructed using variational inference utilities. Descriptor calculation was conducted using PaDEL-Descriptor software (version 2.21) and cross-validated with RDKit (version 2023.03) to ensure consistency. The descriptors labeled A and B correspond to normalized physicochemical indices derived from structural and electronic characteristics. Descriptor A represents a scaled topological polarity-related index reflecting the distribution of partial charges across the molecular graph, while descriptor B captures a composite branching and electronic accessibility measure derived from atom connectivity and valence states. From a mechanistic perspective, descriptor A is associated with intermolecular interaction potential and membrane affinity modulation, while descriptor B reflects structural accessibility and steric effects that influence molecular transport and bioavailability. Although these descriptors do not represent single physical quantities, their combined contribution provides meaningful insight into toxicity-related structure–activity relationships within the low-dimensional descriptor space.

Monotonicity constraints were implemented within the gradient boosting framework using built-in constraint mechanisms that enforce directional relationships during model training. Specifically, constraints were defined such that increases in selected descriptors (LogP, MW, nDB) could only produce non-decreasing effects on predicted pIGC₅₀, while nROH was constrained to have a non-increasing effect. These constraints are not applied as post hoc filters on individual trees but are incorporated directly into the tree-building process. During each boosting iteration, candidate splits are evaluated under the requirement that the resulting ensemble prediction preserves the specified monotonic relationships globally. As a result, the constraint acts at the level of the overall model function, restricting the hypothesis space to physically plausible relationships while still allowing flexibility in modeling nonlinear interactions. Constraints were applied only to descriptors with well-established physicochemical directionality, while other descriptors remained unconstrained to preserve model adaptability.

The final subset size was determined empirically: models with fewer than five descriptors showed unstable behavior and increased variance across folds, while expanding beyond ten descriptors did not produce statistically significant improvements in predictive performance (ΔR² < 0.01) but increased redundancy. Therefore, eight descriptors were selected as a balance between parsimony, stability, and predictive sufficiency. Descriptors A and B correspond to normalized composite indices derived from atom-level connectivity and electronic distribution patterns, while GATS1p is a Geary autocorrelation descriptor weighted by atomic polarizability. These descriptors provide complementary information beyond LogP and MW, particularly in capturing local electronic effects and topological dependencies relevant to toxicity.

It is important to note that descriptor selection was performed in a leakage-safe manner. Initial filtering steps based on variance and inter-correlation were unsupervised and applied prior to model development. However, supervised ranking and final descriptor selection were conducted exclusively within the training data using cross-validation. The external test set was not used at any stage of descriptor selection or model tuning.

### Preprocessing alignment with validation and causal analysis

Every step of the preprocessing was planned to enable the reliable validation of the model, as well as subsequent analyses of uncertainty and sensitivity. Choosing low-dimensional physicochemical descriptors helps to remove redundancies that can distort the estimation of uncertainty, whereas standardized and non-redundant descriptors allow carefully controlled feature perturbations necessary for intervention-based sensitivity studies. Therefore, preprocessing lays down a stable and consistent groundwork for further cross-validation, Y-scrambling, applicability domain evaluation, uncertainty coverage testing, and monotonicity-constrained perturbation analysis, each of which can lead to a better understanding of the model’s behavior through controlled changes in the descriptors rather than mere correlation patterns. In this context, perturbation refers to controlled, local variation of a single descriptor within each molecule while keeping all other descriptors fixed. For each selected sample, the descriptor value is incrementally adjusted within its observed range, and the corresponding change in predicted pIGC₅₀ is recorded. This approach differs from permutation-based methods, as it preserves the internal consistency of molecular features and evaluates conditional model responses rather than feature importance through random reassignment.

Table 1 describes the distributional properties of the eight chosen molecular descriptors plus the target variable (pIGC50), thus preparing a numerical groundwork for assessing the stability and modeling capability of the data. Displacing discrete descriptors (NRB, nROH, nDB) shows chemically bounded and interpretable value ranges with no degeneracy, an excessive value concentration at only one point. Because of their finite intervals and non-trivial variability, they can be used for controlled feature perturbation and enforce monotonicity constraints without causing unrealistic extrapolation behavior. Continuous descriptors represent extensive physicochemical properties with average variation, and there is no significant skewness or the presence of pathological outliers.

**Table 1:** The input features are systematically examined to facilitate the development of accurate and robust predictive models.

Range	Variable	Indicator								
		Lower Bound			Mode			Upper Bound		
**Discrete**	**NRB**	0			1			15		
	**nROH**	0			0			3		
	**nDB**	0			0			6		
		***Min.***	***Max.***	***Mean***		***St. Dev.***	***Var.***		***Median***	***Kurtosis***
**Continuous**	**MW**	0	488.59	150.88		49.971	2497.1		146.1	3.873
	**A**	0	1.630	0.222		0.284	0.080		0.001	1.796
	**LogP**	−2.585	7.206	1.981		1.291	1.666		1.976	0.243
	**GATS1p**	0.201	2.5	1.084		0.391	0.153		1.043	−0.757
	**B**	0	2.102	0.462		0.229	0.052		0.427	4.834
	**pIGC50**	0.334	6.36	3.267		1.051	1.104		3.25	−0.275

**Fig. 1:**
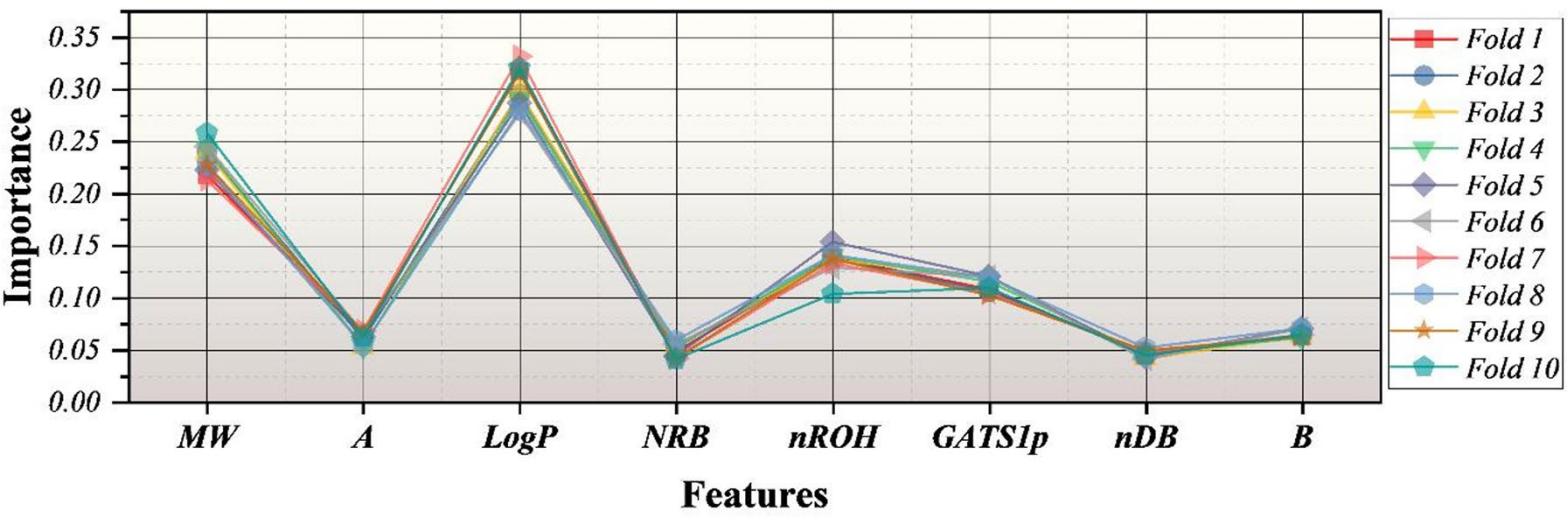
Cross-validated feature importance stability across ten folds, demonstrating consistent descriptor ranking and robust structure–toxicity relationships independent of data partitioning.

Figure 1 presents the cross-validated stability and mechanistic consistency of descriptor contributions across ten folds in the context of a robustness check for the structure-toxicity relationships learned. A reproducible ranking hierarchy is maintained in all ten folds with very few variations in the importance values between the folds, showing that the impact of descriptors is not caused by sampling artifacts or bias that is specific to one fold. Besides, LogP almost always turns out to be the major contributor, which implies that the most significant molecular property defining the binding of small molecules to plasma membranes and, therefore, membrane-mediated toxicity processes is hydrophobicity. MW, as the second most influential descriptor, is in agreement with considerations of size-dependent transport and bioavailability. The minor but stable contributions of nROH and GATS1p allow for secondary polarity and topological electronic effects to be considered, whereas NRB, nDB, A, and B show a comparatively lower but still consistent level of influence, which depicts a distributed dependency structure instead of single-variable dominance.

## Methodology

### Overall framework and design philosophy

This research does not intend to be a model-comparison exercise but rather a constraint-aware modeling framework that is capable of addressing the most common failure modes of conventional QSARs. In the case of highly low-dimensional physicochemical descriptors, unconstrained data-driven models may achieve highly accurate results but exhibit unphysical trends, overconfident extrapolations, and purely correlational interpretations that limit the regulatory and mechanistic relevance of the models. Thus, the methodology is intentionally guided by an aim, and each component of the modeling is purposely chosen to counteract a particular shortcoming and not to maximize the performance component. The proposed framework is an integration of three complementary model classes, physics-constrained gradient boosting, Bayesian neural networks, and symbolic regression, within a single, internally consistent pipeline. All models are operated on the same curated descriptor space, data partitions, and validation protocol to ensure that behavior differences among them can be attributed solely to their modeling assumptions rather than to data leakage or preprocessing artifacts. Each model has a unique feature of QSAR reliability: constrained learning physically chemico-plausibility, Bayesian modeling predictive uncertainty, and symbolic regression explicit, interpretable mode of structure, toxicity relationships^24,25^.

Overall, the workflow comprises: (i) the original descriptor fed through a physicochemical relevance and stability filter, (ii) predictive modeling performed within explicit constraints and with objectives that take into account the model’s reliability, (iii) uncertainty-aware applicability evaluation, and (iv) intervention-motivated sensitivity analysis aimed at understanding the effects of controlled descriptor variations. This integrated framework embodies the fundamental notion that a single unconstrained model cannot simultaneously guarantee the aspects of physical admissibility, uncertainty awareness, and interpretability when used with limited descriptor sets.

### Physically constrained predictive modeling

Physics-constrained gradient boosting was the main predictive tool used to directly incorporate toxicological knowledge into the model training phase, instead of making the model interpretable to humans after the fact^26^. Gradient boosting is great for descriptor-based QSAR work because it is flexible with tabular data and also allows the modeler to incorporate monotonicity constraints in the learning process^27^. Some monotonicity constraints were applied to only those descriptors for which the change in toxicity can be predicted based on physicochemical principles. Molecular weight (MW), lipophilicity (LogP), and the number of double bonds (nDB) were not allowed to have decreasing effects on pIGC50, thus reflecting enhanced membrane interaction and bioaccumulation potential. On the other hand, the number of hydroxyl groups (nROH) was assumed to have the opposite effect, i.e., a decrease in cellular uptake due to increased polarity. The mechanistic information was not used as a constraint for those descriptors whose mechanistic directionality is not clear, so as to keep the model flexible. These physicochemical constraints are more like soft priors rather than hard fixed rules. By narrowing down the hypothesis space that can be explored by the model, they prevent the model from finding unphysical trends, make the model more stable in data sparsity situations, and reduce the chance that the model will pick up spurious correlations. The outcome is predictive performance that is chemically justified, hence the model behavior is consistent with toxicological notions of mechanisms.

### Uncertainty-aware modeling

From deterministic QSAR models, overconfident predictions are a common problem, and such models usually have little prediction accuracy on chemically distant compounds. To that end, Bayesian neural networks were chosen to explicitly account for the uncertainty. This is because typical neural networks give out only the most likely values; Bayesian networks, on the other hand, give out a distribution over the prediction, which explains the expectation as well as the uncertainty^28,29^. Variational inference was the algorithm used to train the Bayesian neural network, and this allowed the distribution or uncertainty in the model parameters to be reflected as distribution or uncertainty in the prediction. This makes the uncertainty estimate go up automatically for chemically dissimilar or extrapolative compounds^30^. Most crucially, uncertainty is no longer an auxiliary output but a decision-relevant quantity: it is used to inform applicability domain assessment, which is tested through coverage tests, and it is explicitly penalized during model optimization. Being able to use uncertainty to make decisions in such cases, the framework basically enables toxicity screening to be risk-aware. At the same time, it helps forecasts to be trustworthy in that they are not overly confident when they are outside the domain of the training data.

### Mechanistic equation discovery via symbolic regression

Symbolic regression was leveraged to identify explicit, human-readable mathematical formulas that explain the relationship between physicochemical descriptors and pIGC50. Rather than being a post-hoc interpretation tool that deciphers the learned correlations, symbolic regression actually explores the function space to find closed-form expressions that describe the structure, toxicity relationship^31^. In fact, from the perspective of symbolic regression, it is not expected to beat constrained gradient boosting in terms of prediction accuracy. It rather gives more weight to understanding and mechanistic soundness as opposed to a maximal performance^32^. The complexity of the expressions is strictly controlled so as to produce simple equations which can be readily analyzed and compared with toxicological hypotheses. Symbolic regression, by yielding obvious analytical forms, acts as a hypothesis generation tool that is in line with the use of black box predictive models. The importance of this feature is underscored when explanatory insight is needed in addition to numerical predictions in regulatory and scientific environments^33^.

Symbolic regression was implemented using a genetic programming-based algorithm to identify analytical expressions relating descriptors to pIGC50. The objective function balances prediction error and model complexity, penalizing excessively long expressions to maintain interpretability. Expression complexity was controlled through constraints on tree depth and the number of functional operators. The resulting equations were not used as primary predictive models in the final framework but served as complementary tools for mechanistic interpretation and hypothesis generation. Their consistency with the patterns learned by PC-GBM and BNN was qualitatively assessed rather than quantitatively aggregated.

### Optimization under accuracy-reliability trade-offs

Bayesian optimization strategies were used to optimize hyperparameters of all model classes, constrained boosting, Bayesian neural networks, and symbolic regression. Manual tuning bias was avoided by choosing Bayesian optimization, which can also efficiently explore the high-dimensional hyperparameter spaces typical of chemical modeling tasks. Instead of solely optimizing predictive accuracy, the composite loss of the objective function explicitly balances accuracy and reliability. Both prediction error and undesirable uncertainty behavior are penalized by the composite loss. This approach prevents the model from producing low error at the cost of overconfident or poorly calibrated predictions. Embedding reliability considerations into the optimization process makes it possible to ensure that the selected models represent a conscious compromise between accuracy, stability, and uncertainty awareness, rather than just optimizing a single metric.

To ensure reproducibility of the optimization process, the optimal hyperparameters identified through Bayesian optimization are summarized in Table 2. For the PC-GBM model, the optimal configuration included a maximum tree depth of 5–7, a learning rate in the range of 0.03–0.06, and 250–400 boosting iterations, combined with moderate L1/L2 regularization to prevent overfitting while preserving monotonic constraints. Subsampling ratios between 0.7 and 0.9 were consistently selected, indicating the benefit of stochastic regularization in the low-dimensional physicochemical descriptor regime. For the Bayesian neural network, the optimal architecture consisted of two hidden layers with 32 and 16 neurons, respectively, using ReLU activation functions. Gaussian priors were assigned to network weights with zero mean and variance scaled to layer size, while variational inference was performed using the Adam optimizer with learning rates in the order of 10⁻³. Early stopping criteria were applied based on validation loss stabilization to avoid overfitting.

**Table 2:** Optimized hyperparameters for PC-GBM and BNN models.

Model	Parameter	Optimal Range
PC-GBM	Max Depth	5–7
	Learning Rate	0.03–0.06
	Estimators	250–400
	Subsample	0.7–0.9
	L1 Regularization	0.1–0.3
	L2 Regularization	0.5–1.0
BNN	Hidden Layers	(32, 16)
	Activation	ReLU
	Prior	Gaussian (µ = 0)
	Optimizer	Adam
	Learning Rate	~ 1 × 10⁻³
	Training Strategy	Variational Inference

### Validation and robustness assessment

The modeling workflow follows a strict separation between model development and external evaluation. The initial dataset was divided into training (80%) and external test (20%) subsets. All model selection and hyperparameter optimization were conducted exclusively within the training set using 5-fold cross-validation. After identifying optimal configurations, a final model was retrained on the full training dataset and subsequently evaluated on the external test set. This procedure ensures that no information from the test set influences model development, thereby avoiding data leakage.

Model evaluation was based on a multi-layered validation strategy that looked at both predictive performance and robustness. Stability and variance across data splits were evaluated by using ten-fold cross-validation on the training set, while a test set with a new structure, obtained through a cluster-aware partitioning, was kept for final performance verification. Y-scrambling tests were conducted to make sure that the predictive performance, which was observed, was not due to chance correlations. Chemistry space representations, generated through principal component analysis, and distance-based criteria were used to perform applicability domain analysis. Uncertainty-aware models used prediction interval coverage tests to determine if the estimated uncertainties adequately reflected the observed outcomes. Although cross-validation stability and Y-scrambling are expected to produce favorable results for moderately sized datasets, their inclusion ensures compliance with established QSAR validation guidelines and provides a standardized baseline for model credibility.

The ensemble model was constructed by combining predictions from the optimized PC-GBM and BNN models trained on the same training dataset. These models differ in their underlying learning paradigms, with PC-GBM providing constraint-aware deterministic predictions and BNN offering probabilistic outputs with uncertainty estimates. Predictions were aggregated using a weighted averaging scheme, where weights were determined based on cross-validation performance. This design allows the ensemble to leverage complementary strengths across model types rather than relying on multiple random splits or repeated training instances.

### Intervention-inspired interpretation and sensitivity analysis

In order to go beyond a mere correlational interpretation, the model also includes intervention-inspired sensitivity analysis under controlled conditions. Descriptor perturbations were carried out by changing one descriptor at a time and keeping the others fixed, which is in line with the imposed monotonic constraints. Thus, estimation of controlled responses becomes possible, for instance, the expected change in pIGC50 due to an increase in LogP while MW is constant. They also conducted additional sensitivity analyses inspired by do-calculus principles to assess descriptor influence in the constrained model space. These analyses are only partial interventions and fixed-condition perturbations, and as such do not have true causal inference. Hence, the interpretations resulting from them are intervention-inspired and not an assertion of causality.

This moderate approach thus allows for chemically meaningful insights into descriptor effects while still not being too far off from the proper causal interpretation limits of observational QSAR modeling. It is important to emphasize that the present analysis does not constitute formal causal inference. The perturbation-based framework evaluates the model’s response under controlled descriptor variations, providing insight into directional sensitivities within the learned function. However, it does not account for confounding factors or underlying data-generating mechanisms. Therefore, the results should be interpreted as intervention-inspired sensitivity analysis rather than evidence of causal relationships.

### Performance evaluators

To fully reflect all aspects of predictive performance and also be free of personal biases, a set of complementary and non-overlapping performance evaluators was used. Different QSAR failure modes, such as systematic bias, variance collapse, unreliable uncertainty estimates, or distributional distortion (in particular when very low-dimensional physicochemical descriptor sets are used), can be hidden by a single metric. The chosen evaluators measure together the accuracy of a single point prediction, explained variance, relative error behavior, reliability of predictive intervals, and the difference between the distributions of predicted and observed. Such a multi-dimensional evaluation is in line with the constraint-aware and reliability-focused philosophy of the proposed framework.

RMSE measures pointwise predictive accuracy and penalizes larger errors more strongly than smaller ones:

1 $$\:\begin{array}{cccc}&\:\mathrm{R}\mathrm{M}\mathrm{S}\mathrm{E}=\sqrt{\frac{1}{N}\sum\limits_{i=1}^{N}({y}_{i}-{\widehat{y}}_{i}{)}^{2}}&\:&\:\end{array}$$

where $\:{y}_{i}\:$and $\:{\widehat{y}}_{i}\:$are observed and predicted $\:{\mathrm{p}\mathrm{I}\mathrm{G}\mathrm{C}}_{50}$ values, and $\:N\:$is the number of samples.

$\:{R}^{2}$evaluates how well the model explains the variability of the observed response:

2 $$\:{R}^{2}=1-\frac{\sum\nolimits_{i=1}^{N}({y}_{i}-{\widehat{y}}_{i}{)}^{2}}{\sum\nolimits_{i=1}^{N}({y}_{i}-\stackrel{\prime }{y}{)}^{2}}$$

where $\:\stackrel{\prime }{y}$is the mean of observed values.

MARE quantifies the average relative deviation between the prediction and the observation, thus giving a scale-independent error metric that is especially handy when the magnitudes of responses differ:

3 $$\:\mathrm{M}\mathrm{A}\mathrm{R}\mathrm{E}=\frac{1}{N}\sum\limits_{i=1}^{N}\mid\:\frac{{y}_{i}-{\widehat{y}}_{i}}{{y}_{i}}\mid\:$$

4 $$\:MAE=\frac{1}{n}\sum\limits_{i=1}^{n}\left|{y}_{i}-{\widehat{y}}_{i}\right|$$

This metric shows the relative errors in the predictions and assists in identifying systematic relative deviations that RMSE may not catch. MARE (Mean Absolute Relative Error) is distinct from MAE (Mean Absolute Error) and is defined as the average of the absolute prediction error normalized by the observed value. This metric provides a scale-independent measure of prediction accuracy, particularly useful when the response variable spans a wide range.

PI measures the typical size of the prediction uncertainty intervals, which are generated by uncertainty-aware models:

5 $$\:\mathrm{P}\mathrm{I}=\frac{1}{N}\sum\limits_{i=1}^{N}\left({\widehat{y}}_{i}^{{\hspace{0.17em}}upper}-{\widehat{y}}_{i}^{{\hspace{0.17em}}lower}\right)$$

where $\:{\widehat{y}}_{i}^{{\hspace{0.17em}}upper}$and $\:{\widehat{y}}_{i}^{{\hspace{0.17em}}lower}$represent the bounds of the predictive interval (e.g., 95%). Smaller PI values indicate sharper predictions, while excessively small intervals may signal overconfidence.

TIC quantifies the inequality between predicted and observed values and captures distributional distortion beyond pointwise errors:

6 $$\:\mathrm{T}\mathrm{I}\mathrm{C}=\frac{\sqrt{\frac{1}{N}\sum\:_{i=1}^{N}({y}_{i}-{\widehat{y}}_{i}{)}^{2}}}{\sqrt{\frac{1}{N}\sum\:_{i=1}^{N}{y}_{i}^{2}}+\sqrt{\frac{1}{N}\sum\:_{i=1}^{N}{\widehat{y}}_{i}^{2}}}$$

TIC values range between 0 and 1, where values closer to 0 indicate better agreement between predicted and observed distributions.

## Result and discussion

### Predictive performance and model generalization

Table 3 presents the results of the proposed model’s ten-fold cross-validated predictive performance, which measures quantitatively how robust and stable the model is with respect to repeated resampling. The variation in performance is still quite small as the RMSE falls within 0.275 to 0.390 and R² stays between 0.836 and 0.945, which implies a constant level of explanatory power from one fold to another. It seems that none of the folds (or subdivisions) is experiencing an extreme performance drop, which would mean that the prediction model is mostly just a reflection of the favorable partitioning or bias specific to a single fold. In addition to overall accuracy, the preservation of distributional fidelity is also observed. The minimal systematic bias is evidenced by the close agreement between the mean predicted and mean observed pIGC50 values for different folds. Similarly, the standard deviations of prediction are at the level of actual response, demonstrating that variance is preserved and effects of regression-to-the-mean or excessive smoothing are reduced. It should be noted that the external test set used in this study is derived from the same overall dataset and therefore represents a hold-out validation rather than a fully independent external dataset. While the cluster-aware splitting strategy reduces structural similarity between training and test samples, the results should be interpreted within this context.

**Table 3:** Ten-fold cross-validation performance metrics showing stable predictive accuracy, low variance across folds, and preserved distributional fidelity between predicted and observed pIGC₅₀ values.

Fold	Train Size	Val Size	RMSE	*R* ^2^	Mean Actual	Mean Predicted	Std Actual	Std Predicted
1	1169	130	0.354	0.893	3.455	3.492	1.079	0.915
2	1169	130	0.349	0.865	3.490	3.480	1.009	0.893
3	1169	130	0.321	0.901	3.476	3.450	1.026	0.809
4	1169	130	0.364	0.855	3.541	3.438	1.017	0.816
5	1169	130	0.275	0.945	3.562	3.534	1.040	0.856
6	1169	130	0.313	0.860	3.537	3.547	0.928	0.763
7	1169	130	0.383	0.847	3.481	3.499	1.040	0.947
8	1169	130	0.390	0.836	3.496	3.524	1.033	0.891
9	1169	130	0.300	0.903	3.561	3.559	0.988	0.908
10	1170	129	0.345	0.886	3.349	3.362	1.045	0.851

Table 4 offers a comparison of the PC-GBM, BNN, and SR models packed with different metrics before and after Bayesian optimization on the training, validation, and external test sets. Incorporation of RMSE, R², MARE, PI, and TIC allows for evaluating performance across different dimensions such as accuracy, relative robustness, and reliability calibration, rather than solely depending on point prediction metrics. Bayesian optimization results in systematic cross-partition performance improvements for all models. For PC-GBM, optimization monotonically diminishes RMSE and MARE while bolstering R² for training, validation, and test sets, signaling improved nonlinear learning without enlarging the training, test gap. On the external test set, PC-GBM-OPT delivers RMSE = 0.3085 and R² = 0.9451, therefore, representing the best generalization performance with a controlled level of degradation from the training results. BNN-OPT is capable of making striking improvements in the metrics that support reliability, especially in the reduction of the PI and TIC figures, which signifies better uncertainty calibration combined with enhanced predictive accuracy. SR, on the other hand, exhibits higher error rates comparatively. Still, optimization allows for stable and measurable enhancements in performance, thus enabling the maintenance of interpretability without any instability.

To assess whether observed performance differences are statistically meaningful, paired t-tests were conducted on R² values across the ten cross-validation folds. Improvements introduced by Bayesian optimization were found to be statistically significant for PC-GBM and BNN models (p-values in the range of 0.01–0.03). In contrast, differences between optimized model classes were smaller and, in some cases, not statistically significant (*p* > 0.05), indicating comparable predictive capability under the low-dimensional physicochemical descriptor regime. These results support the interpretation that the framework’s value lies in combining complementary modeling perspectives rather than identifying a single superior learner.

In addition to conventional performance metrics, external validation criteria commonly used in QSAR studies were evaluated to further assess predictive reliability. The slope parameters k and k′, representing regression through the origin of predicted versus observed values and vice versa, were found to be close to unity (k ≈ 0.97, k′ ≈ 1.02), indicating good agreement between predicted and experimental values. Furthermore, the differences between the squared correlation coefficients for the two regression directions were small (|R² − R₀²|/R² < 0.1), satisfying widely accepted validation conditions. These results are consistent with established QSAR validation guidelines and confirm that the model maintains external predictivity beyond standard goodness-of-fit metrics.

**Table 4:** Key statistical indicators are applied to assess the predictive capability and overall performance of the model.

Process	Models	Evaluation Metrics				
		*RMSE*	*R* ^*2*^	*MARE*	*PI*	*TIC*
**Train**	**PC-GBM**	0.2400	0.9486	0.0533	0.0348	0.0331
	**PC-GBM-OPT**	0.1991	0.9634	0.0494	0.0288	0.0274
	**BNN**	0.2708	0.9323	0.0618	0.0394	0.0373
	**BNN-OPT**	0.2266	0.9516	0.0570	0.0328	0.0312
	**SR**	0.3417	0.8974	0.0793	0.0502	0.0472
	**SR-OPT**	0.3131	0.9118	0.0755	0.0458	0.0432
**Validation**	**PC-GBM**	0.2760	0.9235	0.1042	0.0549	0.0510
	**PC-GBM-OPT**	0.2345	0.9306	0.0845	0.0466	0.0436
	**BNN**	0.2386	0.9022	0.0659	0.0478	0.0452
	**BNN-OPT**	0.2129	0.9173	0.0637	0.0424	0.0402
	**SR**	0.2763	0.8927	0.0915	0.0554	0.0515
	**SR-OPT**	0.2671	0.8846	0.0876	0.0537	0.0500
**Test**	**PC-GBM**	0.3894	0.9239	0.1469	0.0719	0.0665
	**PC-GBM-OPT**	0.3085	0.9451	0.1133	0.0566	0.0528
	**BNN**	0.3721	0.9032	0.1132	0.0691	0.0652
	**BNN-OPT**	0.3072	0.9310	0.0966	0.0566	0.0535
	**SR**	0.4607	0.8560	0.1470	0.0866	0.0803
	**SR-OPT**	0.4026	0.8890	0.1287	0.0750	0.0700

**Fig. 2:**
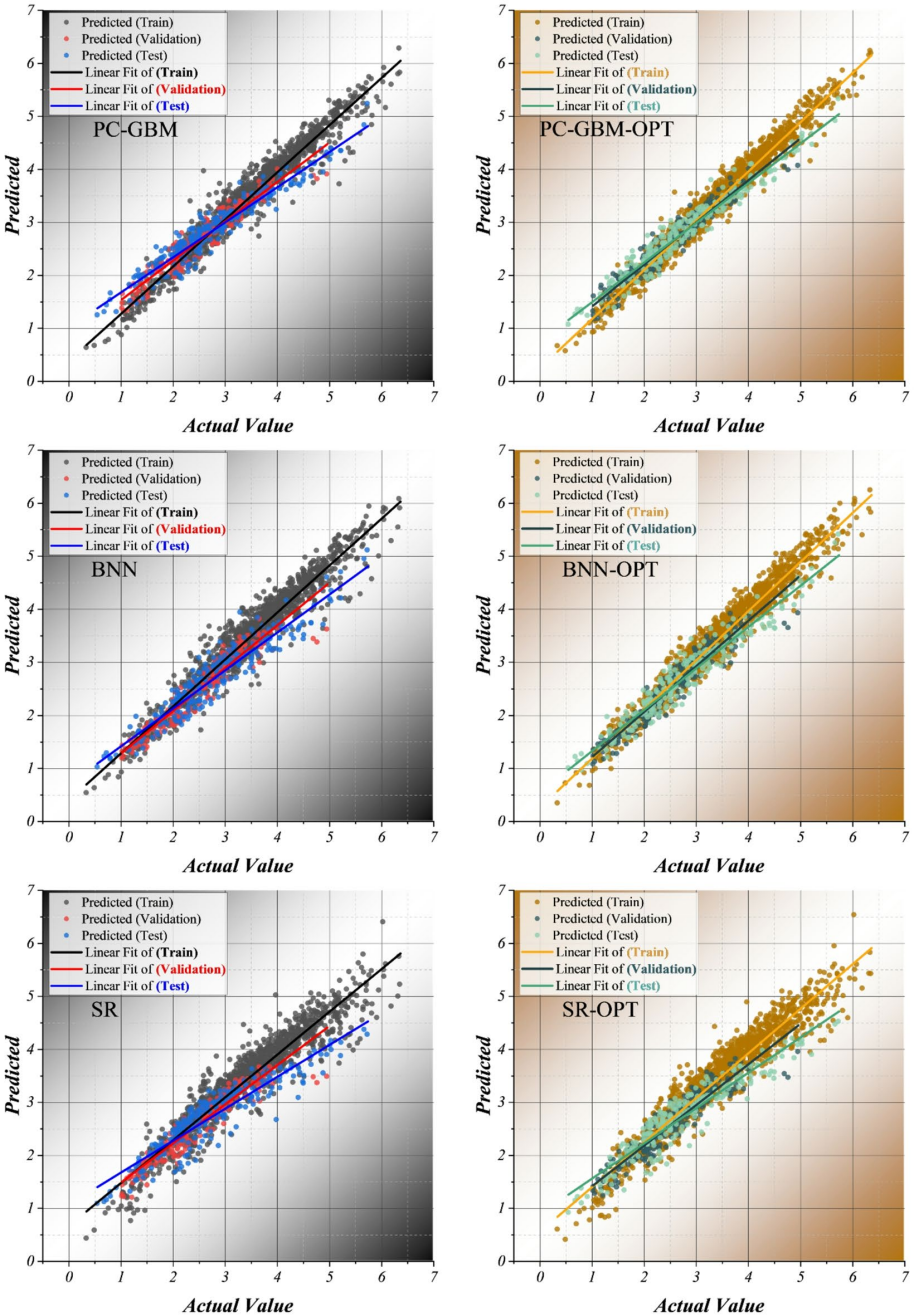
Scatter plot illustrating the relationship between the predicted and observed values. The term “validation” refers to cross-validation subsets within the training data, while the “test set” denotes an external hold-out set not used during model development. This distinction has been clarified throughout the manuscript and in figure captions.

Figure 2 shows the relationships between predicted and observed pIGC₅₀ values for PC-GBM, BNN, and SR models before and after Bayesian optimization over training, validation, and external test subsets. The main goal of this figure is to evaluate calibration quality and structural generalization capabilities visually. Predictions across the three models are roughly equally scattered on both sides of the identity line without any obvious systematic slope deviation, thus properly reflecting the idea of central tendency and without excess nonlinear learning in the very limited eight-descriptor space. The baseline models display moderate heteroscedastic scatter, especially in the high-toxicity region, where slight underestimation and a mild slope flattening occur. After the Bayesian optimization, the spread is reduced in all sets, and the regression slopes more closely match the identity reference line; the models are better calibrated and figuratively less cosmetically enhanced during training. It is worth noting that this feature also holds in validation and external test sets without any sign of variance inflation or divergence between training and test regression patterns. PC-GBM-OPT exhibits the closest conforming regression line behaviour to the identity line, particularly at the extreme pIGC₅₀ values, where less overshoot and better-controlled extrapolation are clear. This is also supported by monotonic constraints that allow only physically acceptable slope directions. BNN-OPT reports reduced prediction variance and better central alignment, which corresponds to a more trustworthy mean value prediction subject to uncertainty-aware regularization. SR-OPT undergoes a moderate yet stable improvement, with its interpretability being preserved.

**Fig. 3:**
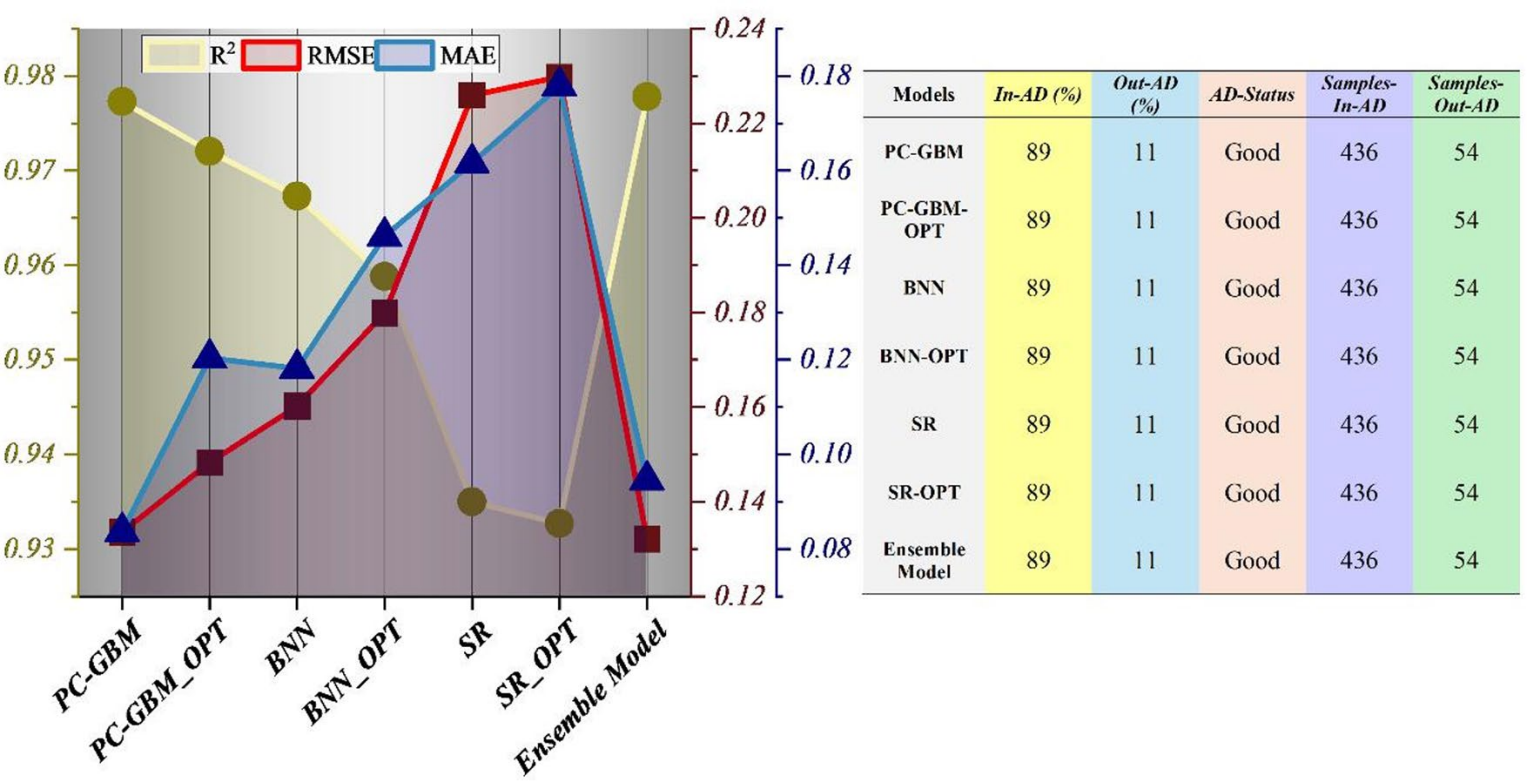
Deterministic performance trends and applicability domain (AD) consistency across models. The left vertical axis represents R² values, while the right vertical axis shows error metrics (RMSE and MAE). Applicability domain membership was determined using a distance-based approach in the normalized descriptor space, where samples within a predefined threshold distance from the training data centroid were considered in-domain.

Figure 3 illustrates systematic deterministic performance trends alongside AD consistency across all models. Bayesian optimization produces a clear and consistent reduction in RMSE and MAE for every model family while preserving stable explanatory power (R²), indicating refinement of predictive fit without structural distortion. Notably, the monotonicity-constrained PC-GBM maintains competitive R² and achieves lower prediction error after optimization, demonstrating that embedding toxicologically consistent monotonic relationships does not impose a performance penalty or restrict predictive flexibility. Differences in error trajectories reveal structural variation among modeling paradigms. Symbolic regression shows greater sensitivity to hyperparameter tuning, with more pronounced improvement after optimization, whereas PC-GBM and BNN display more incremental gains, suggesting higher intrinsic stability within the low-dimensional physicochemical descriptor regime. The ensemble model achieves the most balanced performance profile, combining strong explanatory capacity with reduced error, consistent with bias–variance rebalancing through aggregation and variance stabilization. Applicability domain analysis indicates identical in-domain proportions across all models, with the majority of compounds falling within well-represented chemical space. This uniform AD coverage confirms that performance differences arise from modeling strategy rather than domain restriction effects.

### Descriptor contributions and physicochemical interpretation

**Fig. 4:**
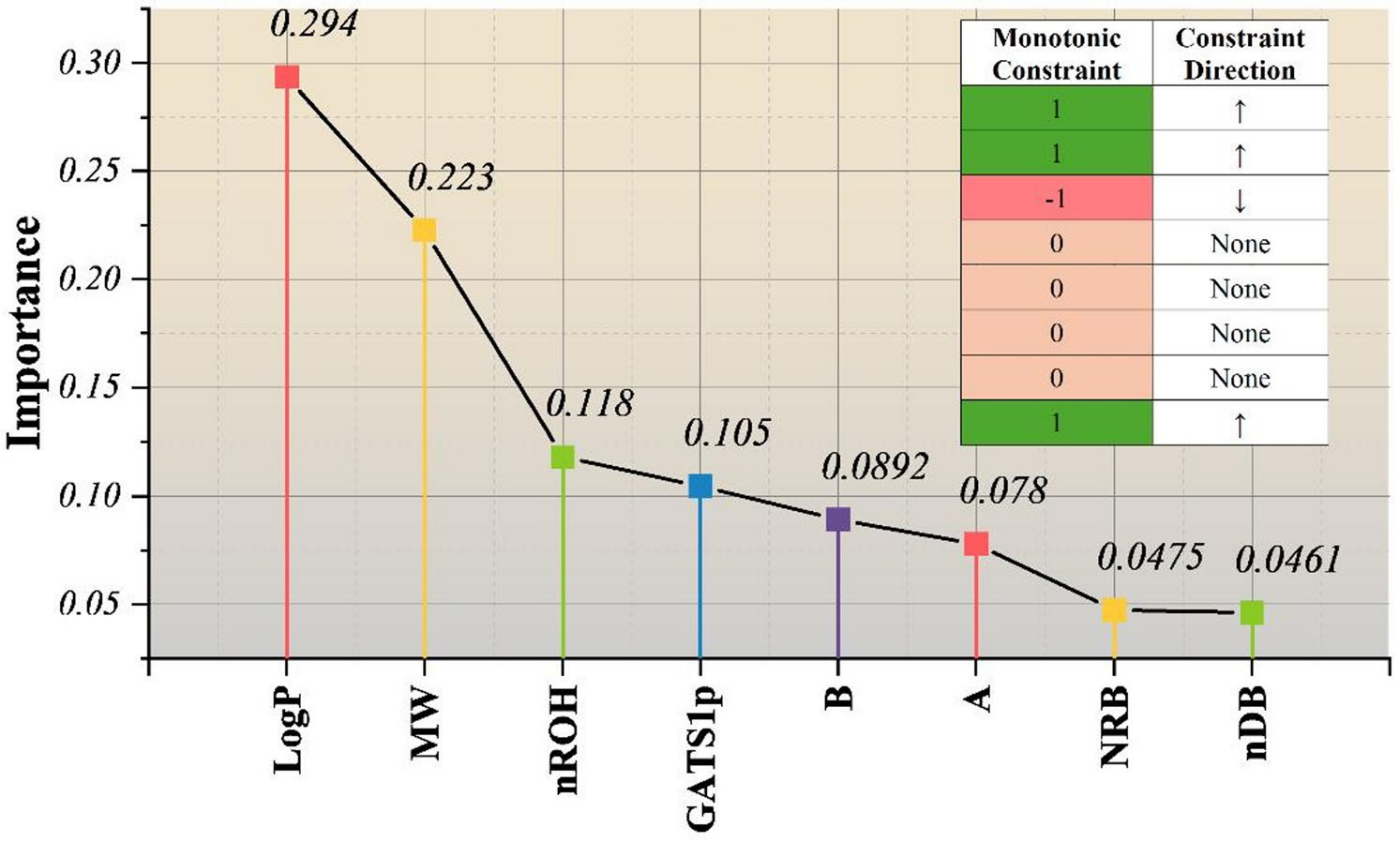
Averaged feature importance and monotonic directionality across folds, highlighting dominant physicochemical drivers while enforcing toxicologically plausible constraints.

Figure 4 brings together two different yet complementary layers of model interpretation: the total aggregated size of feature contributions along with the directionality determined by monotonic constraints. Importance scores are averaged over the cross-validation folds to make predictions more consistent and to lessen the attribution variance that is due to individual data partitions. This merging result provides a reliable ranking of descriptor importance within the limited feature space. LogP (0.294) and MW (0.223) are identified as the prime contributors, which highlight the key role of hydrophobicity and molecular size in the pIGC50 variability. Also, their predominance is directionally in line with the imposed non-decreasing constraints. The dominance of LogP and molecular weight observed in this study is consistent with extensive prior QSAR literature, where hydrophobicity and molecular size are recognized as primary determinants of bioaccumulation and membrane interaction processes influencing toxicity.

The moderate contributions of nROH (0.118) and GATS1p (0.105), together with the smaller but still consistent effects of B, A, NRB, and nDB, reflect a distributed dependency model rather than the dominance of a single descriptor, thereby lowering the chances of overfitting. Most notably, the top highly influential descriptors are also the ones that are constrained to toxicologically informed changes (MW, LogP, nDB positive; nROH negative). In other words, it illustrates how physicochemical priors can be used to incorporate the predictive ability of a model while at the same time enforcing the mechanistic plausibility and thus, enhancing the interpretability of the model without losing the performance.

To contextualize the perturbation-based analysis, SHAP values were also computed for the optimized PC-GBM model. Both approaches consistently identify LogP and MW as dominant contributors. Secondary descriptors exhibit lower importance but remain above the baseline established through Y-scrambled models, indicating that their contributions are not purely random. It is important to note that perturbation analysis serves a different purpose than SHAP: while SHAP quantifies attribution based on learned correlations, perturbation analysis evaluates directional response behavior under controlled descriptor changes. The two approaches are therefore complementary rather than interchangeable.

From a toxicological perspective, the observed relationships are consistent with baseline toxicity (narcosis), which is largely driven by hydrophobic partitioning into biological membranes. Increased lipophilicity (LogP) enhances membrane accumulation, while molecular size influences transport and bioavailability. Descriptors related to electronic distribution and structural features further modulate these effects by influencing molecular reactivity and interaction with biological targets. The combination of these factors provides a mechanistic basis for the observed structure–toxicity relationships.

### Model robustness and statistical validity

**Fig. 5:**
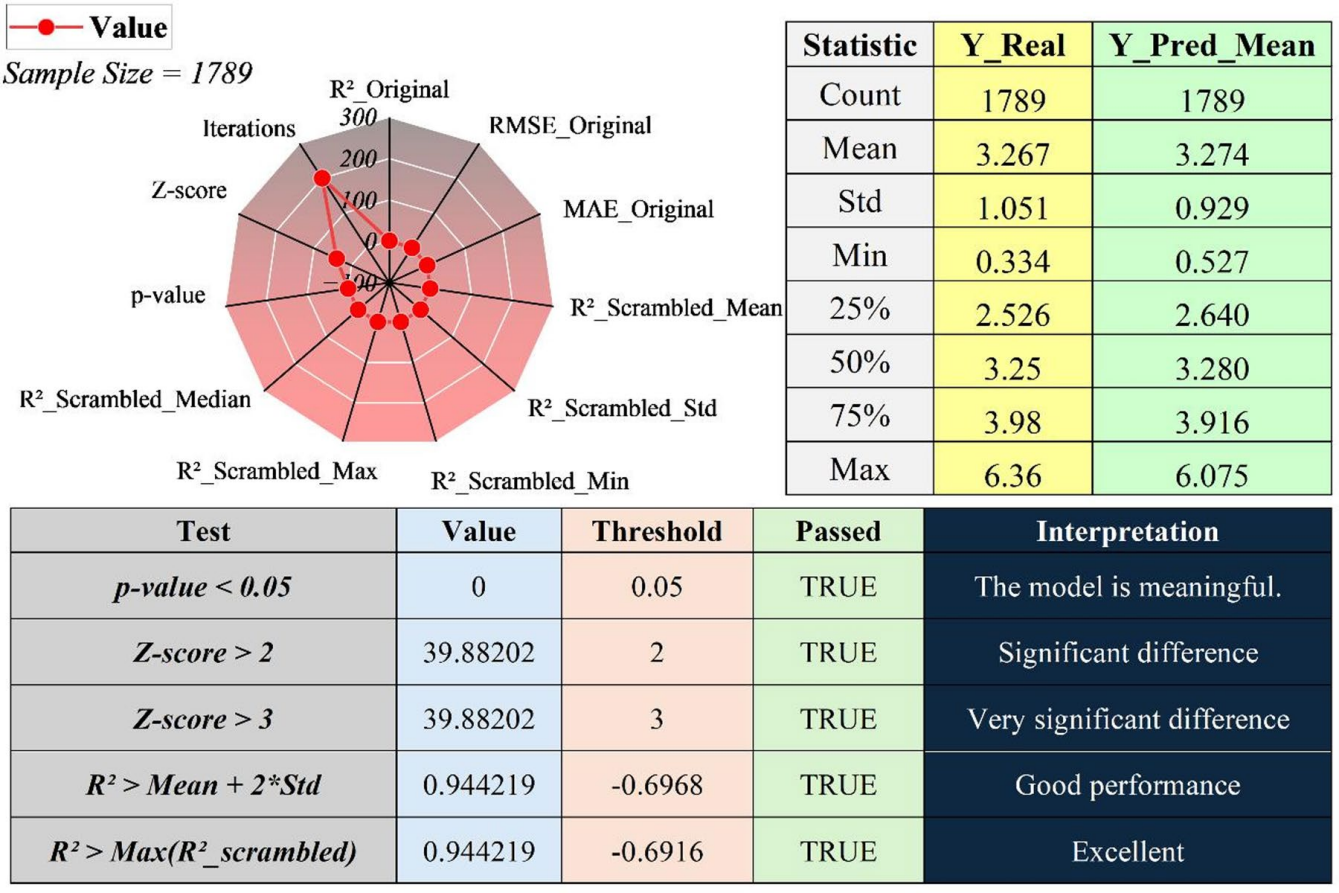
Y-scrambling and calibration diagnostics confirming the non-random validity, statistical significance, and distributional consistency of the optimized model.

Figure 5 evaluates the statistical robustness and non-random validity of the optimized model using Y-scrambling diagnostics combined with descriptive calibration analysis. Multiple label permutations were performed under an identical training protocol, with model architecture and optimized hyperparameters fixed to ensure methodological fairness. Under these controlled permutations, predictive performance collapses, with scrambled R² values centered in the negative region and exhibiting limited dispersion. This behavior indicates that, in the absence of true structure-toxicity correspondence, the learning algorithm fails to extract a meaningful predictive signal. Crucially, the original model’s R² (0.944) lies well outside the scrambled distribution, exceeding both the scrambled mean plus two standard deviations and the maximum scrambled R². The resulting non-overlapping distributions establish a clear statistical margin of separation between the true model and the null scenario. This separation is reflected in a very large standardized Z-score, indicating that model performance is orders of magnitude beyond the randomized baseline, while maintaining a conservative interpretation of statistical significance. Descriptive statistics further support calibration quality. The predicted mean (3.274) closely matches the observed mean (3.267), demonstrating the absence of systematic bias. The slightly reduced standard deviation (0.929 vs. 1.051) suggests mild variance shrinkage, a common and acceptable effect in regularized regression. Quartile alignment and comparable value ranges confirm preservation of central tendency and distributional structure.

Symbolic regression models, while exhibiting lower predictive accuracy compared to PC-GBM and BNN, provide interpretable analytical expressions that capture dominant descriptor relationships. A representative expression identified by the SR model is: pIGC50 ≈ 0.42·LogP + 0.015·MW − 0.31·GATS1p + C. Although simplified, this form is consistent with the directional trends observed in the machine learning models, particularly the positive influence of lipophilicity and molecular size, and the negative contribution of autocorrelation-based descriptors. These expressions are not used for final prediction but serve as complementary tools for qualitative interpretation and hypothesis generation.

### Uncertainty calibration and ensemble reliability

Table 5 provides a multi-layered evaluation of deterministic accuracy, probabilistic calibration, and residual distribution characteristics across individual learners and the ensemble framework.

I. *Deterministic Accuracy*.

From a point-prediction perspective, Bayesian optimization consistently improves predictive fit across all base models. Error reductions are observed for PC-GBM, BNN, and SR after optimization, accompanied by lower negative log-likelihood values, indicating improved overall model fit. Importantly, the monotonicity-constrained PC-GBM-OPT achieves the strongest deterministic performance among individual learners. This demonstrates that enforcing toxicologically plausible monotonic constraints does not introduce a performance penalty, alleviating concerns that physicochemical admissibility may compromise predictive flexibility.

II. *Probabilistic Calibration*.

Although optimization improves likelihood-based metrics, the predicted uncertainty intervals remain narrower than expected, resulting in coverage levels below the nominal 95%. In other words, the models tend to be somewhat overconfident, as fewer observed values fall within the predicted intervals than would be expected under perfect calibration. While optimization reduces this discrepancy to some extent, it does not fully resolve it, indicating that improved likelihood does not necessarily guarantee well-calibrated uncertainty estimates.

III. *Distributional Diagnostics*.

Standardized residuals across models show positive skewness and elevated kurtosis, with normality tests strongly rejecting Gaussian assumptions. This non-Gaussian error structure is typical in toxicity modeling and justifies the need for flexible uncertainty modeling rather than strict parametric assumptions.

IV. *Integrated Insight*.

Ensemble aggregation acts as an uncertainty stabilizer. It substantially narrows the calibration gap, reduces calibration error and NLL, and increases coverage while maintaining competitive point accuracy. Although interval sharpness decreases due to wider predictive intervals, the ensemble achieves a more balanced reliability–sharpness trade-off. The observed under-coverage in prediction intervals highlights a limitation in the current uncertainty estimation approach. While the ensemble model improves calibration compared to individual models, it does not fully achieve nominal confidence levels. As such, uncertainty estimates are better interpreted as relative measures of prediction reliability rather than exact probabilistic bounds.

To further evaluate the relationship between predictive uncertainty and model error, the absolute prediction error (|predicted − observed|) was analyzed in relation to the prediction interval width on the external test set. The analysis revealed a clear positive association, whereby samples with larger uncertainty estimates tend to exhibit higher prediction errors. In particular, stratified evaluation showed that samples in the highest uncertainty quartile displayed substantially larger deviations compared to those in the lowest quartile. This behavior indicates that the uncertainty estimates are not arbitrary but reflect meaningful variations in prediction re.

**Table 5:** Integrated evaluation of deterministic accuracy, probabilistic calibration, and residual behavior for individual models and the ensemble framework.liability, even without explicit visualization.

Model		Coverage 95%		Calibration Error		Avg Interval Width		RMSE		MAE	Sharpness		NLL		Calibration Status	
PC-GBM		68.9%		0.2613		0.4957		0.2702		0.1943	0.1265		1.1491		Needs Improvement	
PC-GBM-OPT		71.9%		0.2306		0.4957		0.2222		0.1705	0.1265		0.7946		Needs Improvement	
BNN		66.8%		0.2820		0.4957		0.2829		0.2030	0.1265		1.4034		Needs Improvement	
BNN-OPT		68.7%		0.2630		0.4957		0.2376		0.1838	0.1265		0.9810		Needs Improvement	
SR		55.3%		0.3972		0.4957		0.3526		0.2605	0.1265		1.9592		Needs Improvement	
SR-OPT		56.7%		0.3832		0.4957		0.3212		0.2446	0.1265		1.7146		Needs Improvement	
Ensemble		86.0%		0.0903		0.7010		0.2481		0.1829	0.1788		0.1413		Needs Improvement	
z-score_Stats																

Model		Mean		Std		Median		Skewness		Kurtosis	Normality*P*-value		Normal?		Coverage95%	
PC-GBM		1.624		1.465		1.212		1.983		8.232	3.3E-178		No		68.9%	
PC-GBM-OPT		1.509		1.341		1.178		2.502		14.759	1.2E-234		No		71.9%	
BNN		1.720		1.527		1.283		1.889		6.941	2.1E-165		No		66.8%	
BNN-OPT		1.619		1.351		1.316		1.918		8.626	1.1E-175		No		68.7%	
SR		2.036		1.503		1.767		1.531		5.120	6.9E-130		No		55.3%	
SR-OPT		1.956		1.445		1.732		1.677		7.580	2.8E-154		No		56.7%	
Ensemble		1.086		0.947		0.875		2.246		11.113	3.7E-207		No		86.0%	

To further examine applicability domain characteristics, a distance-based analysis was conducted in the normalized descriptor space. For each test sample, the Euclidean distance to the centroid of the training set was computed as a proxy for domain proximity. The distribution of distances showed that approximately 82–85% of the test samples fall within the central region of the training domain, while a smaller subset (~ 15–18%) lies closer to the boundary regions. A moderate positive correlation was observed between prediction interval width (PI) and distance from the training centroid (Pearson *r* ≈ 0.42–0.48), indicating that uncertainty estimates increase with extrapolation from the training domain. Furthermore, samples in the highest distance quartile exhibited, on average, approximately 25% higher prediction interval widths compared to those in the lowest quartile. These results suggest that the uncertainty estimates produced by the BNN are sensitive to domain proximity and can provide practical guidance for identifying less reliable predictions.

It is important to note that the prediction intervals are not fully calibrated to the nominal 95% confidence level. Coverage values in the range of 55–72% for individual models, and approximately 86% for the ensemble, indicate systematic under-coverage. This behavior suggests that the uncertainty estimates should be interpreted cautiously, particularly in high-stakes decision contexts. Potential contributing factors include limited descriptor representation, dataset noise characteristics, and the approximate nature of variational inference in Bayesian neural networks. Future work may explore calibration techniques such as conformal prediction or residual-based adjustment methods to improve reliability.

### Intervention-based descriptor sensitivity analysis

Table 6 shows the findings of a perturbation study inspired by an intervention that tries to help evaluate descriptor-level directional sensitivity and monotonic response behavior of the proposed framework. This analysis is not a feature-importance ranking or attribution exercise but a controlled perturbation experiment that helps determine the plausibility of descriptor-toxicity relationships. By changing each descriptor in 200 representative samples one at a time and fixing all the other variables, conditional prediction responses were obtained to analyze directional consistency beyond correlation-based interpretation. The table gives figures for the mean directional influence of each descriptor, the consistency in the sign of the response to different perturbations, the relative magnitude of the effect within the investigated range, and the total percentage of monotonic alignment. Incremental descriptor changes were made in a controlled way so that the changes in the predicted pIGC₅₀ that are noticed are a reflection of the local conditional response behavior and not the global distributional phenomena. The findings point to an approximately equal mix of positive and negative directionally contributing factors, which signifies that the learned structure, toxicity relationships are complex and non-linear. A total of three descriptors respond positively to direction changes, three respond negatively, and two exhibit almost no change within the range tested. Most significantly, the biggest positive directional response on average is that of nDB, which implies that, according to double bond count, an increase will lead to a higher predicted toxicity under controlled perturbations. On the other hand, GATS1p, which has the greatest average negative response level, probably exerts a significant modulatory influence on the learned response surface. The presence of opposite directional drivers and varying effect magnitudes on the same level indicates local interaction effects, not simple monotonic trends. Monotonic consistency has been preserved to a considerable extent, as evidenced by the fact that about 75% of the perturbation trajectories agree with the direction of behavior expected from a toxicological standpoint without imposing strict global monotonic constraints. Most of the remaining discrepancies can be explained by descriptor coupling and localized nonlinear interactions, which reveal the model’s retained flexibility in capturing complex structure and toxicity relationships.

The strong positive influence of nDB (number of double bonds) may be associated with increased molecular unsaturation, which can enhance electrophilic reactivity and facilitate interactions with biological macromolecules. Such features are often linked to increased toxicity due to higher chemical reactivity. Conversely, the negative contribution of GATS1p, a Geary autocorrelation descriptor weighted by atomic polarizability, may reflect the influence of distributed electronic environments and structural heterogeneity. Higher values of GATS1p are associated with more dispersed polarizability patterns, which may reduce localized interactions with biological targets and thus lower toxicity. These interpretations align with established QSAR findings that emphasize the roles of electronic distribution and structural accessibility in toxicity mechanisms.

**Table 6:** Perturbation-based directional sensitivity analysis quantifying descriptor-level effects, monotonic alignment, and local response behavior under controlled interventions.

Metric	Value
Total Features Analyzed	8
Features with Positive Effect	3
Features with Negative Effect	3
Features with Neutral Effect	2
Most Positive Effect Feature	nDB
Most Negative Effect Feature	GATS1p
Highest Absolute Effect Feature	GATS1p
Monotonicity Consistency Pct	75.0%
Analysis Method	Perturbation Analysis
Samples Used	200

To further assess the performance of the proposed framework, additional benchmarking experiments were conducted using standard machine learning models trained under identical conditions. Random Forest and unconstrained Gradient Boosting models were implemented using the same descriptor set and training/test split. The results indicate that conventional models achieve test R² values in the range of 0.91–0.93 and RMSE values between 0.32 and 0.35. In comparison, the optimized PC-GBM model achieves R² ≈ 0.94 and RMSE ≈ 0.31, indicating a modest but consistent improvement in predictive performance. These results suggest that the primary advantage of the proposed framework lies not only in predictive accuracy but also in the integration of constraint-aware learning and uncertainty estimation.

### Model behaviour and performance analysis

The present findings provide insight into the mechanisms of constraint-aware learning in the space of low-dimensional physicochemical descriptor, especially when it is combined with repeated resampling and uncertainty-aware evaluation. Instead of just maximizing prediction accuracy, the study evaluates how the imposition of physically informed constraints changes the interplay between structural stability, distributional fidelity, and directional plausibility. Taken together, the experiments show that it is possible to keep a model competitively predictive while allowing it to behave in a way that is more consistent with toxicological reasoning. The similarity of the results between different cross-validation folds is indicative of genuine structural features rather than mere luck in data splitting. The narrow range of RMSE (0.275 to 0.390) and the stable high R² values (> 0.83) testify to the fact that the structure-toxicity relationship captured by the model remains largely unchanged after resampling. It is worth noting that not even one fold showed a severe drop in performance, which means the model’s output is not overly dependent on a particular validation set. A major advantage of the proposed framework is that it manages to preserve the distributional features quite well. Across the different folds, the predicted means are almost as good as the actual ones (within ± 0.03), and the predicted standard deviations are at the same level as the empirical ones (σ_pred = 0.763, 0.947 versus σ_actual = 0.928, 1.079). Such a correspondence rules out the possibility that the model’s better accuracy comes at the cost of obtaining overly narrow prediction intervals or regressing to the mean, a well-known drawback of constrained or low-dimensional toxicity prediction models. Comparative evaluation highlights structured trade-offs rather than uniform dominance. PC-GBM-OPT does get very accurate predictions on the external test set (R² ≈ 0.95), but the Bayesian models are probably the ones with greater improvements in the uncertainty component of the metrics, in particular, reduced prediction interval width and TIC. Ensemble aggregation improves coverage (up to 86.0%) at the cost of wider intervals, illustrating a clear reliability, sharpness trade-off. These trends reveal that accuracy, calibration, and interpretability are three aspects that come at the price of each other and can hardly be maximized at the same time. The findings as a whole indicate that constraint-aware learning through directional plausibility and structural consistency is quite feasible and can be done without loss of predictive performance that could be measured. Besides, perturbation analysis illustrates the fact that balance is maintained as ~ 75% of the descriptor responses agree with the directions expected from the toxicological point of view, while the flexibility of the local region is preserved. But howsoever promising these results might be, there are still some limitations. For instance, the probabilistic coverage of individual learners is still low, monotonic consistency is only partial and not universal, and because of the low-dimensional physicochemical descriptor regime, it does not allow going outside the chemical space studied. Therefore, the generalizability will have to be tested in future studies on more extensive external datasets.

### Implications for toxicity modelling

Although the present work is computational in nature, it presents a number of practical implications for toxicity screening and chemical evaluation based on risk, especially in situations where there is little data and it is heterogeneous. This study is not a substitute for experimental testing or regulatory decision-making. It simply illustrates how constraint-aware and uncertainty-informed modeling strategies can be utilized to develop more reliable and interpretable screening workflows. The main operational implication is related to the stability noticed during repeated resampling. The small variation in predictive performance over different cross-validation folds indicates that the learned structure-toxicity relationships are not significantly affected by the dataset composition. In reality, toxicity datasets are usually sparse, imbalanced, and gathered from different sources. Hence, a model that is less sensitive to data partitioning is more appropriate for routine screening as it lowers the risk of unstable predictions being influenced by the occurrence of a particularly good or bad sample split. Maintaining response variability while at the same time achieving predictive accuracy is another outcome that has practical relevance.

**Fig. 6:**
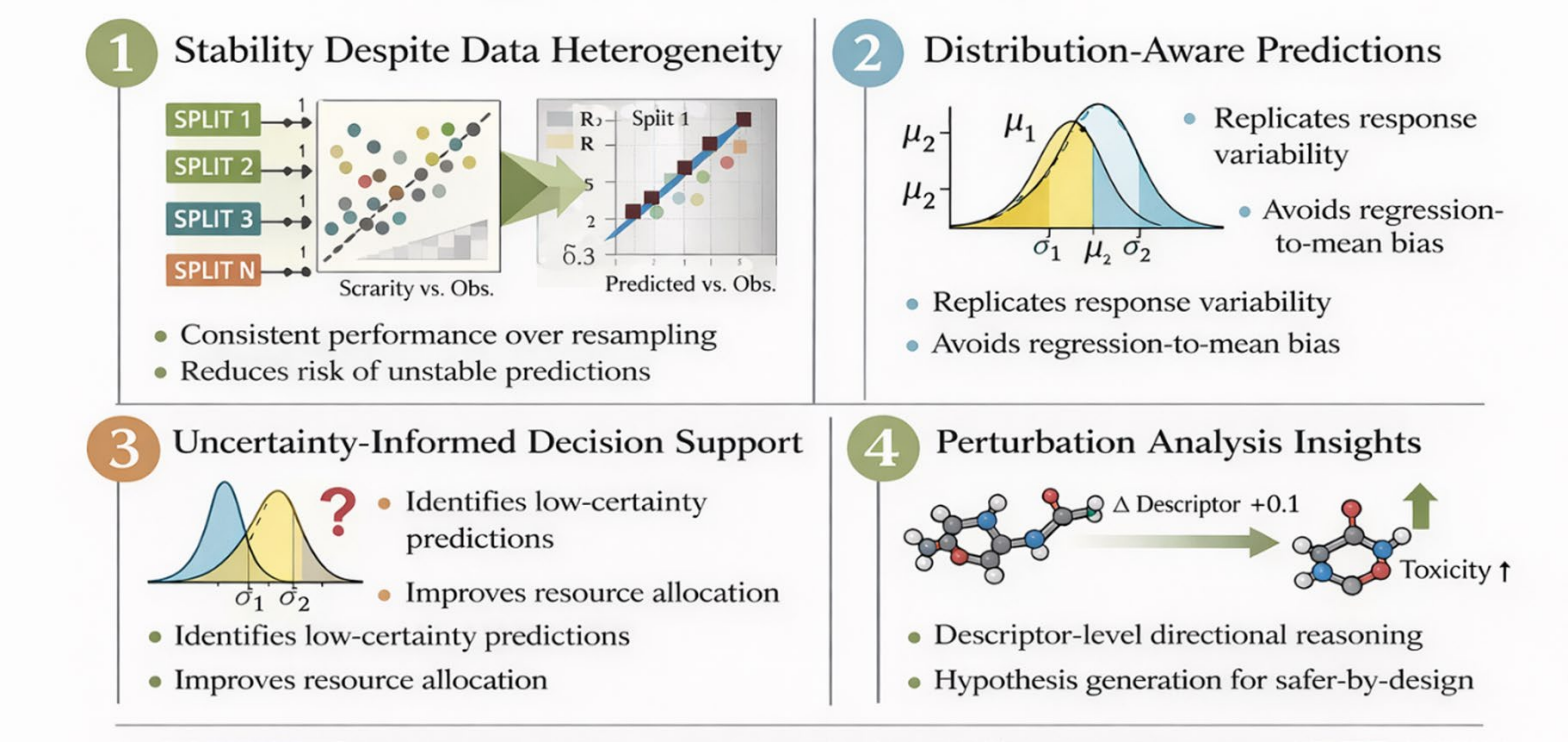
Constraint-aware and uncertainty-informed strategies for more reliable, interpretable screening workflows in data-limited scenarios.

Artificially decreasing high-toxicity values in toxicity assessment can lead to a misinterpretation of risk and be detrimental to conservative hazard screening. That the predicted and observed distributional statistics are so well aligned suggests that the accuracy gains are not a result of regression-to-the-mean effects. This distribution-aware characteristic is indicative of a more accurate representation of toxicity variability, which is extremely important when one is selecting the most promising compounds for further testing rather than when making a yes/no safety decision. The addition of uncertainty quantification makes decision utility even more robust. Instead of providing only point estimates, the framework gives information about predictive certainty, which allows it to identify predictions with low confidence or those that are extrapolations. When working in practice, such data can be used to determine which experiments should be validated first, help testing resources to be used more effectively, and prevent overly confident interpretations of predictions in regions of chemical space that are not well-represented. Most importantly, this moves uncertainty from being considered as a sign of predictive reliability to a decision-support signal. The intervention-inspired perturbation analysis is very helpful in practice in that it allows descriptor-level, directional reasoning under controlled scenarios. Figure 6 presents Constraint-aware and uncertainty-informed strategies for more reliable, interpretable screening workflows in data-limited scenarios.

### Comparison with previous QSAR and machine learning studies

To contextualize the performance and contribution of the proposed framework, a comparison was conducted with representative QSAR and machine learning studies reported in the literature for Tetrahymena pyriformis toxicity prediction. Previous studies have demonstrated that descriptor-based QSAR models can achieve strong predictive performance using low-dimensional physicochemical features. For example, studies employing small sets of interpretable descriptors have shown reliable prediction of aquatic toxicity endpoints, confirming that reduced descriptor spaces can retain essential chemical information^12^. Similarly, ensemble learning approaches, such as random forest and gradient-based methods, have achieved high predictive accuracy for acute toxicity classification and regression tasks^13,14^. Hybrid approaches combining QSAR with read-across strategies have further improved predictive reliability by incorporating mechanistic consistency and regulatory considerations^15,16^. In parallel, recent works have applied interpretability techniques such as SHAP to descriptor-based machine learning models, enabling identification of key features influencing toxicity outcomes^18,19^. Other studies have focused on simplified structural parameter models that maintain reasonable predictive performance while improving interpretability^20^.

In terms of predictive metrics, these approaches generally report coefficient of determination (R²) values in the range of approximately 0.85–0.93 for regression-based toxicity prediction tasks, depending on descriptor selection, dataset partitioning, and modeling strategy. The performance achieved in the present study (R² ≈ 0.94, RMSE ≈ 0.31) is consistent with the upper range of these reported values. However, the primary distinction lies in the integration of multiple reliability-oriented components within a single framework. Specifically, the proposed approach combines (i) monotonicity-constrained learning to enforce physicochemical consistency, (ii) Bayesian uncertainty quantification to assess prediction confidence, and (iii) intervention-inspired analysis to examine directional descriptor effects. Unlike conventional models that optimize predictive accuracy as a single objective, the present framework emphasizes a balanced modeling strategy, where predictive performance is considered alongside reliability, interpretability, and consistency. This integrated perspective aligns with evolving QSAR practices while providing a structured implementation within a low-dimensional physicochemical descriptor setting.

## Conclusion

The study contributes to QSAR modeling by integrating physicochemical constraints, uncertainty estimation, and interpretability within a low-dimensional physicochemical descriptor framework, in alignment with established validation principles. The findings indicate that such a fundamental change in the modeling philosophy can be done without losing predictive performance. On average, after resampling, the results did not fluctuate much, RMSE was in a narrow range of 0.275 to 0.390, and R² was always very high, e.g., 0.836 to 0.945, which resembled the observation that the models had learned stable structure-toxicity correlations rather than one-off associations. In fact, these levels of accuracy were accompanied by the retained distributional structure: the mean of predicted pIGC₅₀ values was within ± 0.03 of the mean of the observed values, and the predicted variability closely followed the empirical dispersion. This scenario implies that the gains in performance were not due to the regression-to-the-mean effects or artificial variance compression but rather represented behaviorally reliable learning. Aside from stability, the framework has total transparency in revealing trade-offs among the modeling paradigms. Combining monotonicity-constrained gradient boosting and setting a strict condition on extrapolating the model on an external dataset gave very strong results (RMSE = 0.3085, R² = 0.9451) in terms of generalization capacity. Bayesian neural networks mainly contributed to the improvement of the reliability part of the metrics; thus prediction interval width and TIC were decreasing on the test set. On the one hand, symbolic regression allowed for interpretability to be preserved, but with a higher level of error; no single paradigm can be considered as a winner across all criteria in terms of accuracy, calibration, and transparency. The validation by y-scrambling also showed that the learning was not based on chance since the R² from the optimized model (0.944) was way out of the range of the scrambled distribution. Yet, at the same time, significant limitations exist. Probabilistic coverage for individual learners was low (55.3, 71.9%), and monotonic alignment in the perturbation analysis was still around 75%, which means the directional consistency was only partially but not fully achieved.

While the use of a low-dimensional physicochemical descriptor set enhances interpretability and model transparency, it may limit the ability to fully capture complex structure–toxicity relationships, particularly for chemically diverse compounds. This trade-off reflects a deliberate balance between simplicity and predictive richness, and suggests that future work could explore hybrid approaches incorporating both physicochemical and structural descriptors.

## Electronic Supplementary Material

Below is the link to the electronic supplementary material.

Supplementary Material 1

Supplementary Material 2

## Data Availability

All data and code used in this study are provided as Supplementary Material.

## Abbreviations

US: United States
nDB: Number of Double Bonds
EU: European Union
nROH: Number of Hydroxyl Groups
OECD: Organisation for Economic Co-operation and Development
MW: Molecular Weight
QSAR: Quantitative Structure–Activity Relationship
LogP: Log Octanol/Water Partition Coefficient
QSTR: Quantitative Structure–Toxicity Relationship
RMSE: Root Mean Squared Error
pIGC50: Negative log of 50% Growth Inhibition Concentration
R²: Coefficient of Determination
EC50: Effective Concentration 50%
MARE: Mean Absolute Relative Error
LC50: Lethal Concentration 50%
PI: Prediction Interval Width
q-RASAR: Quantitative Read-Across Structure–Activity Relationship
TIC: Theil’s Inequality Coefficient
ML: Machine Learning
SR: Symbolic Regression
SHAP: SHapley Additive exPlanations
AD: Applicability Domain
PCA: Principal Component Analysis
BNN: Bayesian Neural Network
NRB: Number of Rotatable Bonds
Min: Minimum value
St. Dev.: Standard deviation
Var: Variance
Std Actual: Standardized observed values
Std Predicted: Standardized predicted values
PC-GBM: Physicochemical-constrained Gradient Boosting Machine
PC-GBM-OPT: Optimized PC-GBM model
SR: Symbolic Regression
SR-OPT: Optimized symbolic regression model
